# Construction of a nomogram prediction model for individualized prediction of the risk of left ventricular diastolic dysfunction in maintenance hemodialysis patients

**DOI:** 10.3389/fcvm.2026.1780590

**Published:** 2026-05-14

**Authors:** Zhoutao Xie, Binhui Pan, Wenwen Hu, Yaqian Cheng, Renban Wang

**Affiliations:** Department of Nephrology, Wenzhou Central Hospital, Wenzhou, Zhejiang, China

**Keywords:** influencing factors, left ventricular diastolic dysfunction, left ventricular hypertrophy, maintenance hemodialysis, nomogram prediction model

## Abstract

**Objective:**

To explore the influencing factors of left ventricular diastolic dysfunction (LVDD) in maintenance hemodialysis (MHD) patients and construct a nomogram prediction model.

**Methods:**

Data was collected from 357 patients who received MHD treatment in our hospital from April 2022 to December 2024. According to a 7:3 ratio, the patients were grouped into a modeling group of 250 cases and a validation group of 107 cases. The modeling group was grouped into LVDD group of 61 cases and non LVDD group of 189 cases based on whether LVDD occurred. Multivariate logistic regression was used to analyze the risk predictive factors of LVDD in MHD patients in the modeling group. R software was used to draw nomograms. The calibration curve and Hosmer-Lemeshow goodness of fit test were used to evaluate the calibration of the nomogram. The receiver operating characteristic (ROC) curve was used to evaluate the discriminative power of the nomogram. Clinical decision curve analysis (DCA) was used to evaluate the clinical utility of nomograms.

**Results:**

Age, left ventricular hypertrophy, hypertension, diabetes, LVMI and hemoglobin were risk predictors of LVDD in MHD patients (*P* < 0.05). The consistency between the predicted values in the calibration curves of the modeling and validation groups and actual observed values was good, with Hosmer-Lemeshaw test *P* = 0.317 and 0.320, and the AUC of the ROC curve was 0.922 and 0.896, indicating good calibration and discrimination ability of the model. When the high-risk threshold probabilities of the modeling group and validation group were within the range of 0.02–0.98 and 0.04–0.86, the net benefit of using a nomogram prediction model to make treatment decisions by clinical physicians was high.

**Conclusion:**

The nomogram prediction model constructed in this study can help clinicians identify LVDD high-risk patients in MHD and improve management strategies.

## 1 Introduction

Cardiovascular disease is a common comorbidity in patients with chronic kidney disease and the leading cause of death in patients receiving maintenance hemodialysis (MHD) (1–3). Left ventricular diastolic dysfunction (LVDD) is highly prevalent among patients with chronic kidney disease, occurring early in the course of heart disease, and progressive diastolic dysfunction is independently associated with a higher risk of mortality (4–6). Early detection and intervention of LVDD can reduce the incidence of cardiovascular events and improve the prognosis of MHD patients. In this study, we aimed to investigate the relevant risk factors for LVDD by integrating baseline data and blood laboratory results of MHD patients, and to evaluate its impact on cardiac function. Furthermore, this study utilized the identified risk factors to construct a nomogram prediction model, aiming for early prediction of LVDD and providing guidance for the clinical diagnosis and functional management of cardiovascular disease in MHD patients.

## 2 Participants and study design

### 2.1 Study subjects

Data were collected from 357 patients receiving MHD treatment at our hospital between April 2022 and December 2024. Patients were divided into a modeling group (*n* = 250) and a validation group (*n* = 107) at a 7:3 ratio, with the modeling group used to construct the nomogram prediction model. This study received approval from the hospital's Medical Ethics Committee.

**Figure 1:**
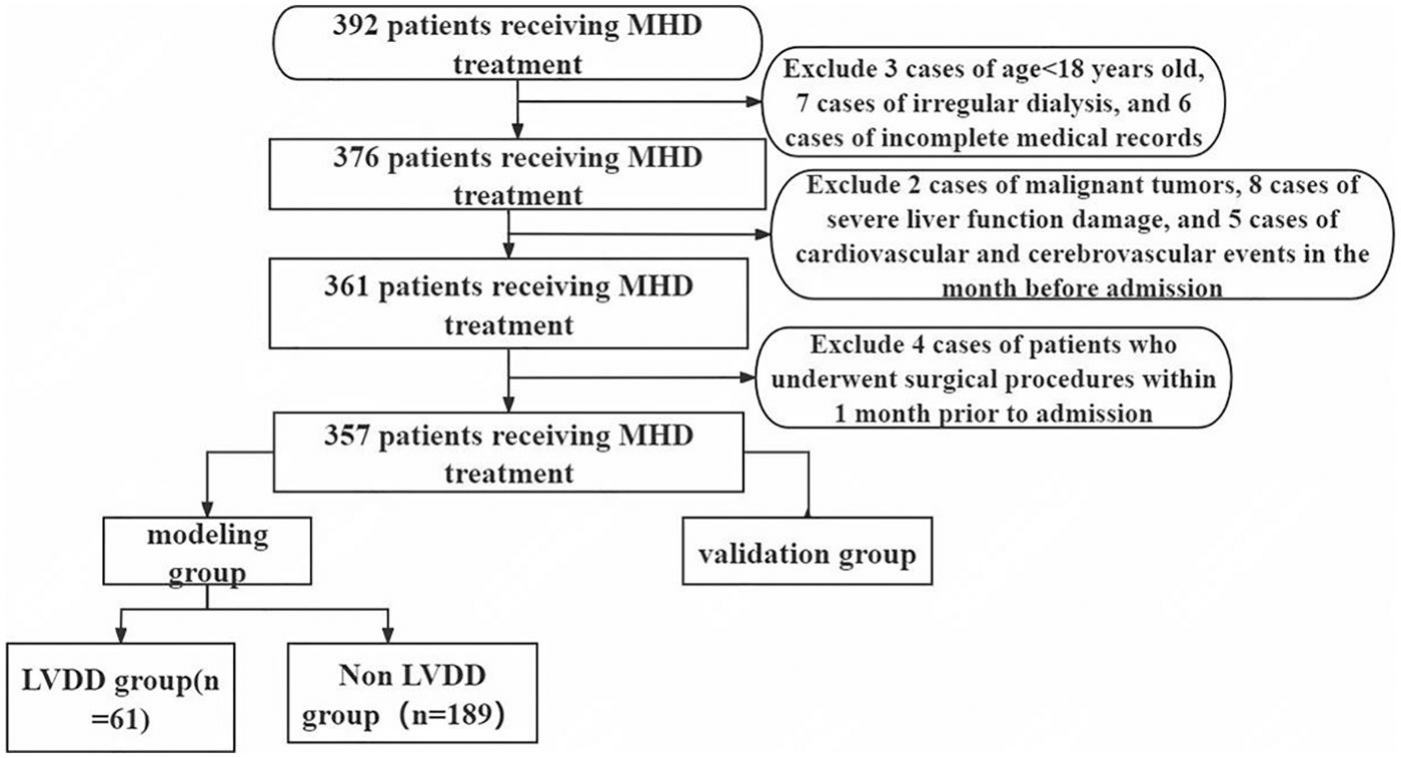
Case collection process diagram.

Inclusion criteria: (1) Age ≥ 18 years; (2) Received regular MHD treatment for more than 3 months, with 3 sessions per week, each lasting 4 h. Exclusion criteria: (1) Incomplete medical records; (2) Presence of malignant tumors or severe liver dysfunction; (3) History of cardio-cerebrovascular events such as arrhythmia, cerebrovascular accident, or heart failure within 1 month prior to admission; (4) History of trauma, acute inflammation, or other surgical treatments within 1 month prior to admission. The case collection flowchart is shown in Figure 1.

### 2.2 Data collection

Clinical characteristic data of MHD patients were collected, including baseline information, dialysis-related data, and blood laboratory examinations. Baseline information included: age, sex, body mass index (BMI), systolic blood pressure, diastolic blood pressure, smoking status, alcohol consumption, left ventricular hypertrophy, hypertension, coronary artery disease, diabetes, and use of angiotensin-converting enzyme inhibitors/angiotensin II receptor blockers (ACEI/ARB). Dialysis-related data included: dialysis vintage, vascular access type. Blood laboratory examinations included(All measurements were obtained at a single time point): brain natriuretic peptide (BNP), cardiac troponin T (cTnT), high-density lipoprotein cholesterol (HDL-C), low-density lipoprotein cholesterol (LDL-C), triglycerides (TG), total cholesterol (TC), creatinine, urea clearance index (Kt/V), calcium, phosphorus, hemoglobin, albumin, uric acid, 25-hydroxyvitamin D3 [25-(OH)D3], intact parathyroid hormone (iPTH), and arteriovenous fistula laterality. Cardiac ultrasound measurements were performed by experienced sonographers on non-dialysis days. Patients were placed in the left lateral decubitus position, and measurements were obtained at rest. Before the examination, patients were instructed to rest in the supine position for at least 10 min and to avoid strenuous activity and caffeine intake that could affect hemodynamics. All parameters were obtained during the same examination session to minimize the potential influence of pre- and post-dialysis volume status changes on diastolic function indices. Measurements included left ventricular end-diastolic diameter (LVDd), interventricular septal thickness (IVST), and posterior wall thickness (PWT). Endocardial borders at end-diastole and end-systole were traced in apical two- and four-chamber views. The tricuspid regurgitation peak velocity (TRV) was measured using continuous-wave Doppler, and the maximum regurgitant velocity was recorded. In this study, TRV was measurable in all patients, and the regurgitation signals were clearly visualized. Left ventricular ejection fraction (LVEF) was calculated using the Simpson method. Left ventricular mass was calculated as: 0.8 × 1.04 × [(LVDd + IVST + PWT)³—LVDd³] + 0.6. Left ventricular mass index (LVMI) was calculated as left ventricular mass/body surface area. All echocardiographic images were independently analyzed by two experienced cardiologists, both of whom were blinded to the patients' clinical data (including laboratory parameters and group allocation). In cases of discrepant measurements, a third senior cardiologist reviewed the images and the average value was recorded. The final diagnosis was based on agreement between the two primary reviewers.

LVDD Diagnosis: According to the 2016 recommendations by the American Society of Echocardiography and the European Association of Cardiovascular Imaging (7), the following four criteria were used: (1) Septal e′ velocity < 7 cm/s or lateral e′ velocity < 10 cm/s; (2) Average E/e′ ratio > 14; (3) Left atrial volume index (LAVI) > 34 mL/m^2^; (4) TRV > 2.8 m/s. Diastolic dysfunction was indicated if two or more criteria exceeded the threshold values. The modeling group was divided into an LVDD group (*n* = 61) and a non-LVDD group (*n* = 189) based on the presence or absence of LVDD. In the LVDD group, septal e′ was (6.2 ± 0.8) cm/s, lateral e′ was (8.1 ± 1.0) cm/s, mean E/e′ was (15.8 ± 2.5), LAVI was (38.5 ± 4.2) mL/m^2^, and TRV was (3.1 ± 0.4) m/s.In the non-LVDD group, septal e′ was (8.5 ± 1.2) cm/s, lateral e′ was (11.2 ± 1.5) cm/s, mean E/e′ was (9.2 ± 1.8), LAVI was (28.6 ± 3.1) mL/m^2^, and TRV was (2.4 ± 0.3) m/s.

### 2.3 Statistical analysis

Statistical analysis was performed using SPSS software (version 25.0). Normally distributed continuous data were expressed as mean ± standard deviation (mean ± SD) and compared using independent samples *t*-tests; Categorical data were expressed as counts (percentages) [*n* (%)] and compared using the chi-square (*χ*²) test; Multivariable logistic regression analysis was used to identify risk predictors for LVDD in the modeling group of MHD patients, with results expressed as odds ratios (OR); A nomogram was plotted based on the logistic regression results using the RMS package in R software; Internal validation was conducted using 1,000 bootstrap resamples;The calibration of the nomogram was assessed using calibration curves and the Hosmer-Lemeshow goodness-of-fit test; The discrimination of the nomogram was evaluated using the receiver operating characteristic (ROC) curve; The clinical utility of the nomogram was assessed using decision curve analysis (DCA). A two-sided *P* < 0.05 was considered statistically significant.

## 3 Results

### 3.1 Comparison of clinical characteristics between modeling and validation groups

There were no statistically significant differences (*P* > 0.05) between the modeling and validation groups in terms of age, sex, BMI, dialysis vintage, systolic blood pressure, diastolic blood pressure, smoking status, alcohol consumption, vascular access type, ACEI/ARB medication use, left ventricular hypertrophy, hypertension, coronary artery disease, diabetes, BNP, cTnT, LVEF, LVMI, HDL-C, LDL-C, TG, TC, creatinine, Kt/V, calcium, phosphorus, hemoglobin, albumin, uric acid, 25-(OH)D3, iPTH, and arteriovenous fistula laterality. See Table 1.

**Table 1:** Comparison of clinical characteristics between modeling group and validation group[*n*(%)/($\bar{x}\pm s$)].

Index	Modeling group(*n* = 250)	Validation group(*n* = 107)	*χ^2^/t*	*P*
Age (years old)			1.492	0.222
≤60	146 (58.40)	55 (51.40)		
>60	104 (41.60)	52 (48.60)		
Gender [*n*(%)]			1.286	0.257
Male	138 (55.20)	66 (61.68)		
Female	112 (44.80)	41 (38.32)		
BMI(kg/m^2^)	22.66 ± 2.75	22.89 ± 2.93	0.710	0.478
Dialysis age (years)	3.61 ± 1.48	3.90 ± 1.42	1.717	0.087
Systolic pressure(mmHg)	146.37 ± 21.79	149.82 ± 22.56	1.356	0.176
Diastolic pressure(mmHg)	83.11 ± 10.85	85.37 ± 11.04	1.794	0.074
Smoke [*n*(%)]	121 (48.40)	62 (57.94)	2.732	0.098
Drink [*n*(%)]	34 (13.60)	18 (16.82)	0.625	0.429
Vascular access [*n*(%)]			1.737	0.187
Venous catheterization	88 (35.20)	30 (28.04)		
Arteriovenous fistula	162 (64.80)	77 (71.96)		
Left ventricular hypertrophy [*n*(%)]	113 (45.20)	53 (49.53)	0.565	0.452
Hypertension [*n*(%)]	154 (61.60)	75 (70.09)	2.350	0.125
Diabetes [*n*(%)]	43 (17.20)	11 (10.28)	2.795	0.095
BNP(pg/mL)	487.59 ± 120.74	499.76 ± 128.93	0.855	0.393
cTnT(ng/L)	46.52 ± 12.98	44.53 ± 13.82	1.301	0.194
LVEF (%)	60.48 ± 6.94	62.01 ± 6.79	1.921	0.056
LVMI(g/m^2^)	116.64 ± 22.90	120.85 ± 24.46	1.559	0.120
HDL-C(mmol/L)	1.03 ± 0.22	1.05 ± 0.19	0.819	0.414
LDL-C(mmol/L)	2.24 ± 0.52	2.13 ± 0.47	1.883	0.060
TG(mmol/L)	1.26 ± 0.36	1.32 ± 0.34	1.467	0.143
TC(mmol/L)	4.19 ± 1.09	4.24 ± 1.13	0.393	0.695
Creatinine(*μ*mol/L)	862.73 ± 235.18	882.54 ± 237.69	0.727	0.468
Kt/V	1.61 ± 0.28	1.55 ± 0.27	1.875	0.062
Ca(mmol/L)	2.25 ± 0.19	2.29 ± 0.22	1.736	0.083
P(mmol/L)	1.77 ± 0.45	1.74 ± 0.48	0.566	0.572
Hemoglobin(g/L)	96.90 ± 18.75	100.42 ± 22.37	1.531	0.127
Albumin(g/L)	40.11 ± 3.97	40.75 ± 4.62	1.327	0.185
Uric acid(μmol/L)	450.30 ± 90.57	464.92 ± 89.49	1.402	0.162
25-(OH)D3	21.27 ± 5.94	22.08 ± 5.73	1.193	0.234
iPTH	274.66 ± 62.03	287.35 ± 65.76	1.739	0.083
Arteriovenous fistula side [*n*(%)]			0.695	0.405
Left	233 (93.20)	97 (90.65)		
Right	17(6.80)	10(9.35)		

MHD patients were divided into a modeling group of 250 cases and a validation group of 107 cases based on a 7:3 ratio; BNP, B-type natriuretic peptide; cTnT, cardiac troponin T; LVEF, left ventricular ejection fraction; LVMI, left ventricular mass index; HDL-C, high-density lipoprotein cholesterol; LDL-C, low-density lipoprotein cholesterol; TG, triglycerides; TC, total cholesterol;25-(OH)D_3_, 25-hydroxyvitamin D3; iPTH, intact parathyroid hormone; ACEI/ARB, angiotensin converting enzyme inhibitor/angiotensin II receptor blocker.

### 3.2 Comparison of clinical characteristics between non-LVDD and LVDD groups in the modeling group

Within the modeling group, there were no statistically significant differences (*P* > 0.05) between the two groups in terms of sex, BMI, dialysis vintage, systolic blood pressure, diastolic blood pressure, smoking status, alcohol consumption, coronary artery disease, vascular access type, ACEI/ARB medication use, BNP, cTnT, LVEF, HDL-C, LDL-C, TG, TC, creatinine, Kt/V, calcium, phosphorus, albumin, uric acid, uric acid, 25-(OH)D3, iPTH, and arteriovenous fistula laterality. In the modeling group, patients in the LVDD group had higher rates of age >60, left ventricular hypertrophy, hypertension, diabetes, and higher LVMI compared to the non-LVDD group, while hemoglobin levels were lower than in the non-LVDD group (*P* < 0.05). See Table 2.

**Table 2:** Comparison of clinical characteristics between non LVDD group and LVDD group in the modeling group [*n*(%)/($\bar{x}\pm s$)].

Index	Non LVDD group(*n* = 189)	LVDD group(*n* = 61)	*χ^2^/t*	*P*
Age (years old)			14.224	0.000
≤60	123 (65.08)	23 (37.70)		
>60	66 (34.92)	38 (62.30)		
Gender [*n*(%)]			1.182	0.277
Male	108 (57.14)	30 (49.18)		
Female	81 (42.86)	31 (50.82)		
BMI(kg/m^2^)	22.57 ± 2.84	22.92 ± 2.56	0.857	0.393
Dialysis age (years)	3.52 ± 1.48	3.90 ± 1.50	1.738	0.083
Systolic pressure(mmHg)	145.07 ± 20.12	150.41 ± 22.63	1.747	0.082
Diastolic pressure(mmHg)	82.51 ± 10.94	84.95 ± 10.71	1.522	0.129
Smoke [*n*(%)]	95 (50.26)	26 (42.62)	1.078	0.299
Drink [*n*(%)]	24 (12.70)	10 (16.39)	0.536	0.464
Vascular access [*n*(%)]			0.206	0.650
Venous catheterization	68 (35.98)	20 (32.79)		
Arteriovenous fistula	121 (64.02)	41 (67.21)		
Left ventricular hypertrophy [*n*(%)]	71 (37.57)	42 (68.85)	18.224	0.000
Hypertension [*n*(%)]	102 (53.97)	52 (85.25)	19.073	0.000
Coronary artery disease	61 (32.28)	27 (44.26)	2.905	0.088
Diabetes [*n*(%)]	22 (11.64)	21 (34.43)	16.812	0.000
Application of ACEI/ARB drugs			3.082	0.079
No	117 (61.90)	30 (49.18)		
Yes	72 (38.10)	31 (50.82)		
BNP(pg/mL)	485.19 ± 126.50	494.98 ± 117.23	0.535	0.593
cTnT(ng/L)	45.74 ± 12.06	48.95 ± 13.66	1.749	0.082
LVEF (%)	60.89 ± 5.17	59.22 ± 8.15	1.881	0.061
LVMI(g/m^2^)	110.52 ± 23.75	135.62 ± 22.87	7.241	0.000
HDL-C(mmol/L)	1.04 ± 0.22	1.01 ± 0.24	0.905	0.366
LDL-C(mmol/L)	2.23 ± 0.56	2.29 ± 0.48	0.752	0.453
TG(mmol/L)	1.25 ± 0.37	1.31 ± 0.35	1.116	0.266
TC(mmol/L)	4.18 ± 1.14	4.22 ± 1.06	0.242	0.809
Creatinine(μmol/L)	846.48 ± 225.72	913.06 ± 252.94	1.944	0.053
Kt/V	1.60 ± 0.22	1.63 ± 0.45	0.696	0.487
Ca(mmol/L)	2.25 ± 0.19	2.27 ± 0.20	0.706	0.481
P(mmol/L)	1.78 ± 0.42	1.75 ± 0.50	0.462	0.644
Hemoglobin(g/L)	102.43 ± 20.94	79.76 ± 16.74	7.696	0.000
Albumin(g/L)	40.39 ± 4.15	39.26 ± 3.54	1.913	0.057
Uric acid(μmol/L)	446.75 ± 91.32	461.28 ± 89.24	1.086	0.278
25-(OH)D_3_(ng/mL)	21.64 ± 6.35	20.13 ± 5.15	1.686	0.093
iPTH(pg/mL)	27 (1.45 ± 60.29)	284.62 ± 67.33	1.441	0.151
Arteriovenous fistula side [*n*(%)]			0.625	0.429
Left	178 (94.18)	55 (90.16)		
Right	11(5.82)	6(9.84)		

The modeling group was divided into LVDD group (61 cases) and Non LVDD group (189 cases) based on whether LVDD occurred; BMI, body mass index; BNP, B-type natriuretic peptide; cTnT, cardiac troponin T; LVEF, left ventricular ejection fraction; LVMI, left ventricular mass index; HDL-C, high-density lipoprotein cholesterol; LDL-C, low-density lipoprotein cholesterol; TG, triglycerides; TC, total cholesterol; 25-(OH)D_3_, 25-hydroxyvitamin D3; iPTH, intact parathyroid hormone; ACEI/ARB, angiotensin converting enzyme inhibitor/angiotensin II receptor blocker.

### 3.3 Multivariable logistic regression analysis of risk predictors for LVDD in MHD patients in the modeling group

Variables with *P* < 0.05 in the univariate analysis from Section 3.2 were included in the multivariable analysis. Variable assignments are shown in Table 3. Multivariable logistic regression analysis identified risk predictors for LVDD in MHD patients. The results showed that age (OR = 3.195), left ventricular hypertrophy (OR = 5.610), hypertension (OR = 6.088), diabetes (OR = 3.436), LVMI (OR = 1.047), and hemoglobin (OR = 0.928) were risk predictors for LVDD in MHD patients (*P* < 0.05). See Table 4.

**Table 3:** Variable assignment table.

Variable	Assignment
Age	1 = >60 years old, 0 = ≤60 years old
Left ventricular hypertrophy	1 = Yes, 0 = No
Hypertension	1 = Yes, 0 = No
Diabetes	1 = Yes, 0 = No
LVMI	Continuous variable
Hemoglobin	Continuous variable
Dependent variable	1 = LVDD, 0 = non LVDD

LVMI, left ventricular mass index.

**Table 4:** Multivariate logistic regression analysis of risk predictive factors for LVDD in MHD patients.

Influence factor	β	SE	Waldχ^2^	OR	95% CI	*P*
Age	1.162	0.446	6.791	3.195	1.334–7.653	0.009
Left ventricular hypertrophy	1.725	0.458	14.197	5.610	2.288–13.760	0.000
Hypertension	1.806	0.516	12.274	6.088	2.216–16.723	0.000
Diabetes	1.234	0.521	5.619	3.436	1.238–9.533	0.018
LVMI	0.045	0.010	22.389	1.047	1.027–1.066	0.000
Hemoglobin	−0.075	0.014	30.579	0.928	0.903–0.953	0.000
Constant	−2.831	1.500	3.560	0.000	–	0.000

LVMI, left ventricular mass index.

### 3.4 Construction of a nomogram model for predicting LVDD in MHD patients

**Figure 2:**
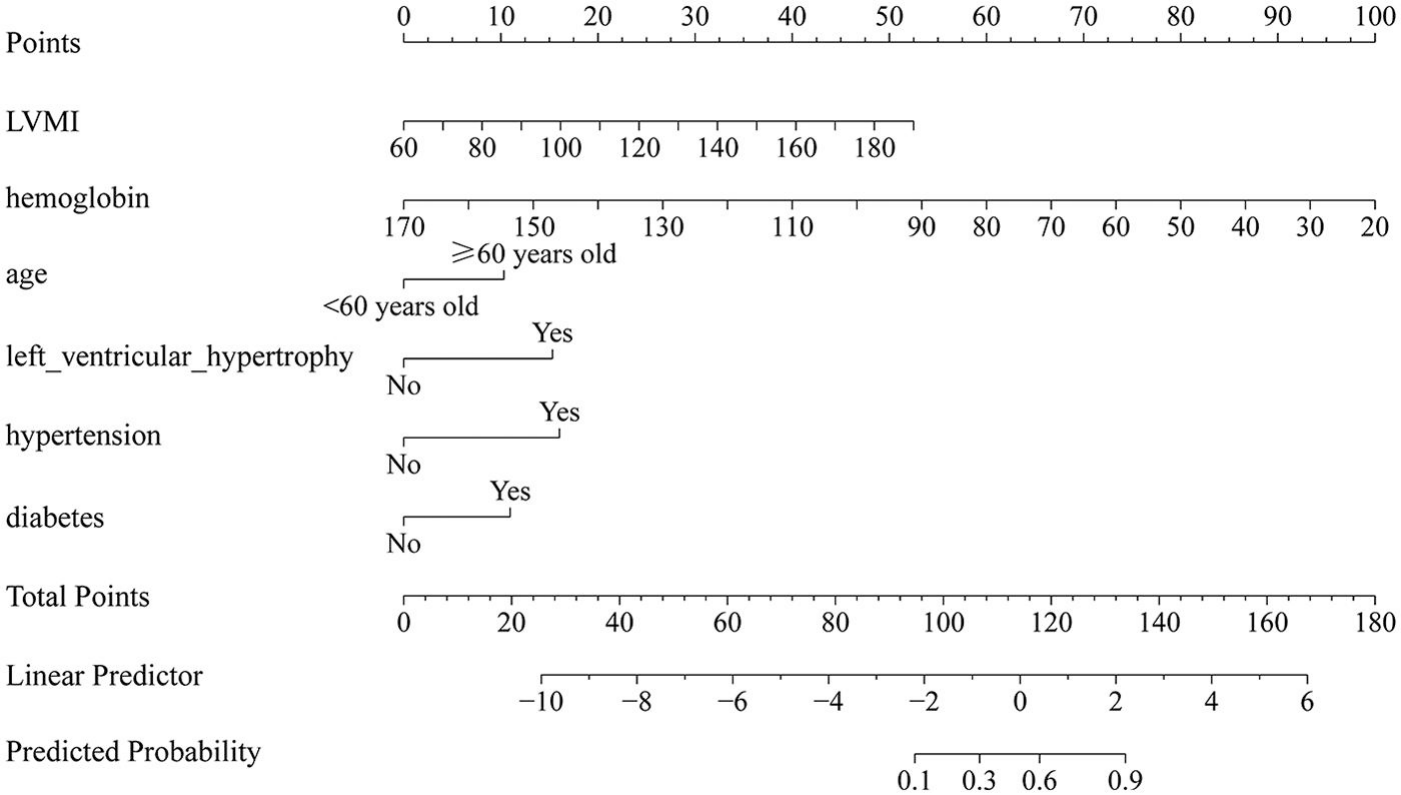
Nomogram for predicting LVDD in MHD patients.

Note: Each risk prediction factor corresponds to a risk point, and a vertical line can be drawn up from the corresponding values of each factor to the “Points” axis to obtain their respective scores. All scores are added up to obtain the total score, and a vertical line can be drawn down from the “Total Points” axis to the “Predicted Probability” axis to obtain the patient's risk of LVDD; If an MHD patient has an LVMI of 120 g/m^2^ (24.86 points), hemoglobin of 110 g/L (39.72 points), age ≤ 60 years old (0 points), left ventricular hypertrophy (15.04 points), hypertension (15.18 points), and no diabetes (0 points), the total score is 94.80 points, and the corresponding Predicted Probability is 0.48, that is, the risk of LVDD in the MHD patient may be 48%.

A nomogram model was constructed based on the risk predictors for LVDD in MHD patients: age, left ventricular hypertrophy, hypertension, diabetes, LVMI, and hemoglobin. See Figure 2. Each risk predictor corresponds to a risk point score. The score for each factor can be obtained by drawing a vertical line directly upwards from the corresponding value of the factor to the “Points” axis. The sum of all scores yields the total score. A vertical line drawn downwards from the “Total Points” axis to the “Predicted Probability” axis provides the patient's risk of developing LVDD.

### 3.5 Validation of the nomogram prediction model in the modeling and validation groups

**Figure 3:**
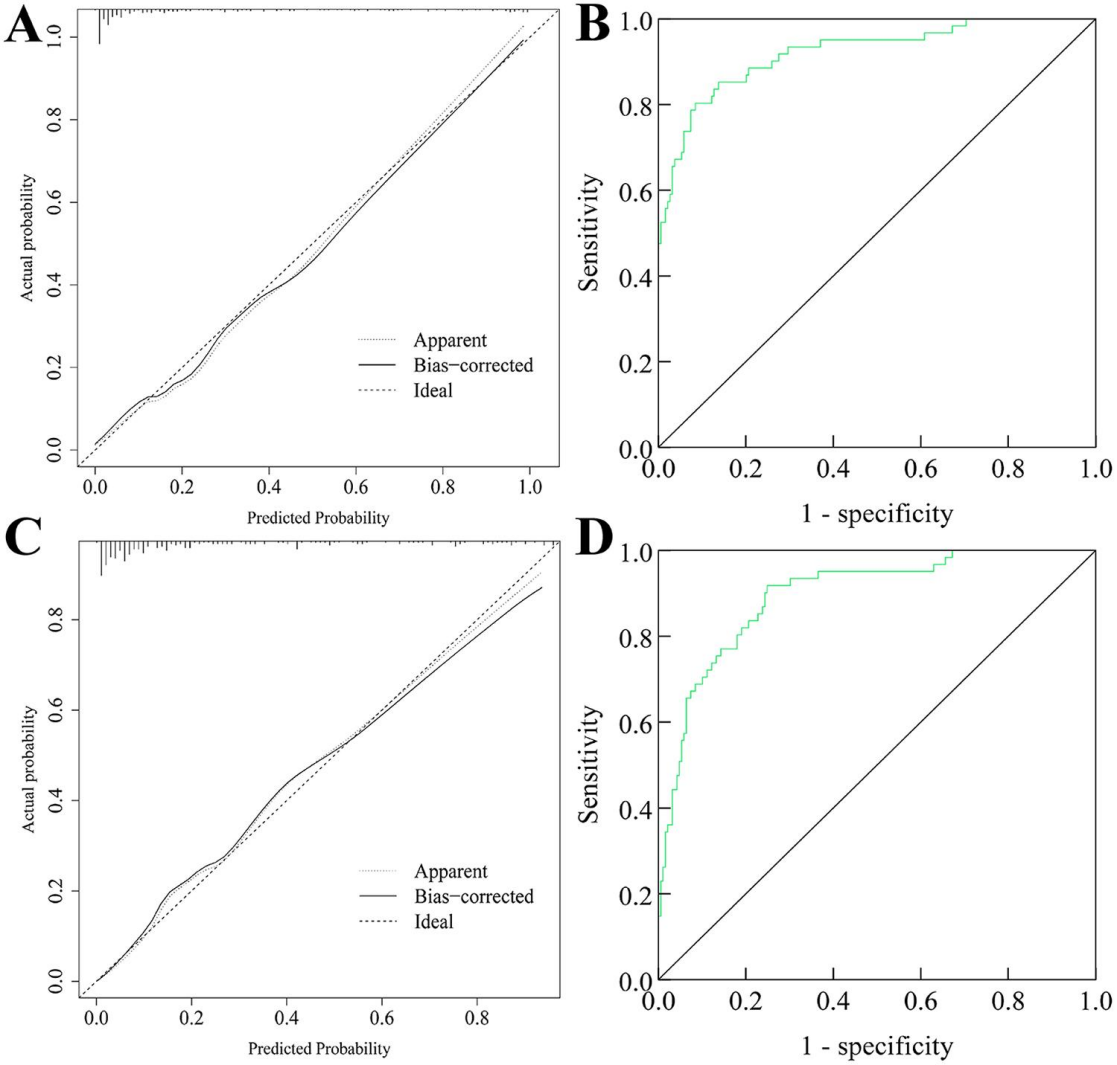
The validation of the nomogram prediction model in the modeling group and the validation group. **(A)** Calibration curve of modeling group; **(B)** ROC curve of modeling group; **(C)** Calibration curve of validation group; **(D)** ROC curve of validation group.

The nomogram prediction model was validated using calibration curves and ROC curves. The calibration curve for the modeling group (Figure 3A) indicated good agreement between the model's predicted values and the actual observed values, with the Hosmer-Lemeshow test yielding *P* = 0.317 (not significant). The AUC of the ROC curve (Figure 3B) was 0.922 (95% CI: 0.8800.964). The calibration curve for the validation group (Figure 3C) also showed good agreement between predicted and observed values, with the Hosmer-Lemeshow test yielding *P* = 0.320 (not significant). The AUC of the ROC curve (Figure 3D) was 0.896 (95% CI: 0.8510.940), indicating good calibration and discrimination ability of the model.

### 3.6 Assessment of the clinical application of the nomogram prediction model

**Figure 4:**
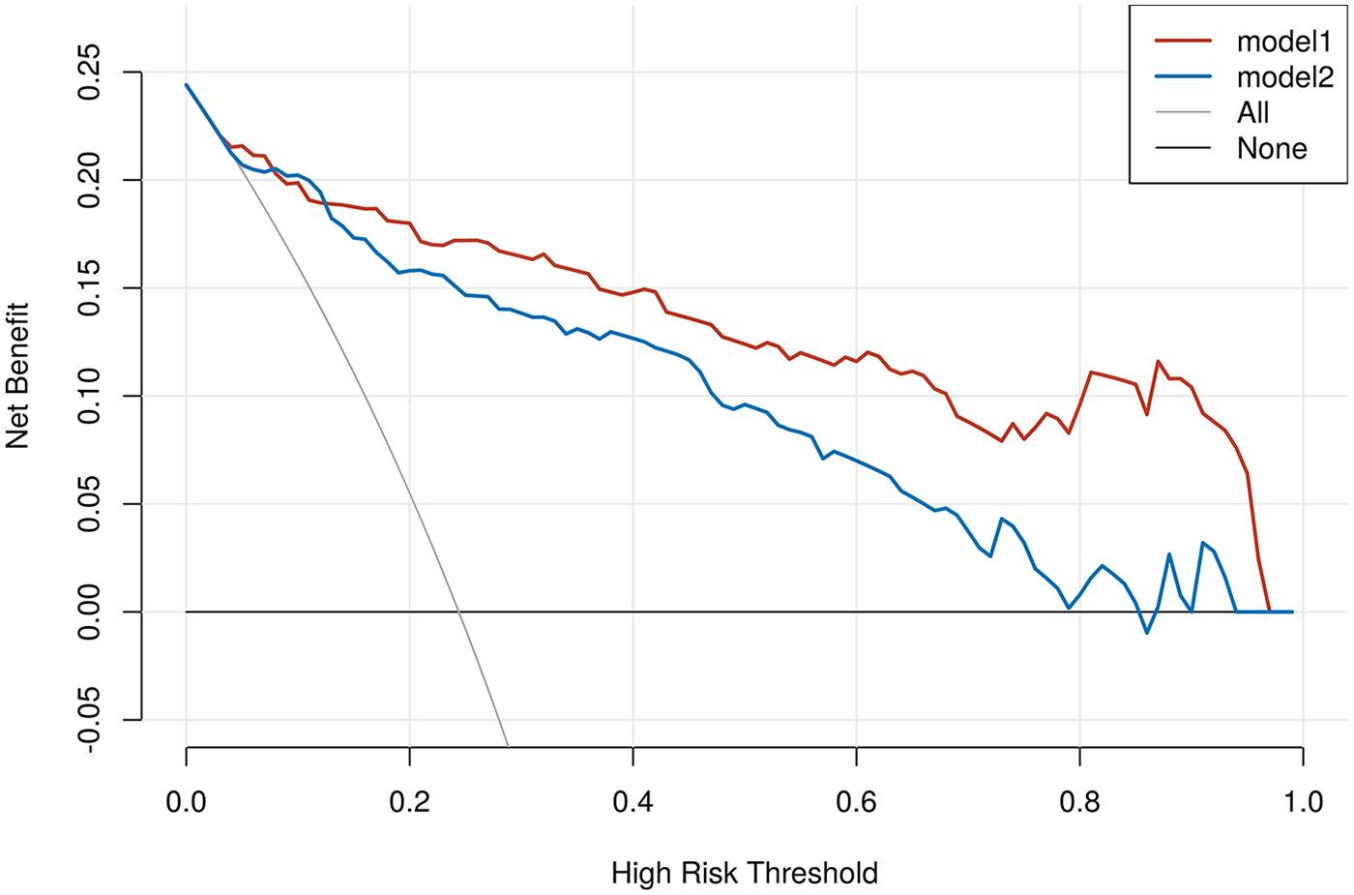
DCA curves for the nomogram prediction model. model 1 is modeling group; model 2 is validation group; All line represents the hypothesis that all patients receive treatment; None line represents the hypothesis that all patients do not receive treatment.

Decision curve analysis (DCA) was used to verify the clinical utility of the model. The results showed that within the high-risk threshold probability ranges of 0.020.98 for the modeling group (model 1) and 0.040.86 for the validation group (model 2), clinicians using the nomogram prediction model developed in this study would achieve more net benefit compared to treating all patients (All line) or treating no patients (None line). See Figure 4.

## 4 Discussion

This study found that age, left ventricular hypertrophy, hypertension, diabetes, LVMI, and hemoglobin were significantly associated with LVDD in MHD patients. Utilizing these factors, a nomogram was developed to predict the risk of LVDD occurrence in patients receiving MHD. The nomogram prediction model was found to improve overall prediction accuracy to a certain extent, demonstrating high calibration, discrimination, and clinical utility.

The cardiovascular event prediction model for hemodialysis populations developed by Li et al. (8) indicated that age, albumin, and history of cardiovascular disease are related to the occurrence of cardiovascular events, which is consistent with the results of this study. Increasing patient age was positively correlated with the risk of cardiovascular events, with an OR value of 3.195. As patients age, the decline in bodily functions may lead to vascular wall stiffening and decreased cardiac function, thereby increasing the risk of LVDD occurrence; furthermore, left ventricular wall thickness and fibrosis increase with age, which forms the pathophysiological basis for structural cardiac changes and electrophysiological dysfunction (9). In this study, left ventricular hypertrophy was also an independent risk factor for LVDD in MHD patients (OR = 5.610), similar to the findings of Lei et al. (10), whose results also identified age >60 years as a risk factor for LVDD in MHD patients. Left ventricular hypertrophy is one of the most common myocardial changes in patients with end-stage kidney disease, often associated with myocardial fibrosis and diastolic dysfunction (11). Various pathological factors in end-stage kidney disease patients readily lead to cardiac fibrosis, structural abnormalities, and thickening of intramural arteries; the adaptive response to pressure and volume overload causes left ventricular hypertrophy (12), which can further progress to LVDD. Bansal et al. (13) found that hypertension is a significant risk factor for cardiovascular disease in patients undergoing MHD treatment; this study similarly found that hypertension is associated with LVDD in MHD patients. Volume overload, arterial stiffness, endothelial dysfunction, and increased activity of the sympathetic nervous and renin-angiotensin-aldosterone systems in MHD patients can all trigger hypertension (14). Hypertension, in turn, increases cardiac load, leading to myocardial hypertrophy, reduced ventricular compliance, and impaired diastolic function. Furthermore, in this study, diabetes increased the risk of LVDD onset in MHD patients (OR = 3.436). Mathew et al. (15) similarly found that diabetic hemodialysis patients had a 7.66 times higher risk of diastolic dysfunction. MHD patients with diabetes experience further metabolic disturbances, leading to increased myocardial cell inflammation, fibrosis, apoptosis, and metabolic abnormalities, making them prone to concentric left ventricular hypertrophy and subsequently LVDD. The results of this study showed that increased LVMI is an independent risk factor for LVDD in MHD patients (OR=1.047). In the results of Luo et al. (16), age, diabetes, and LVMI were all predictors of heart failure in hemodialysis patients, similar to this study. LVMI is a key marker of cardiovascular stress, especially in dialysis patients where cardiovascular stress can induce symptoms like palpitations and chest pain, exacerbate existing heart conditions such as causing blood pressure fluctuations, increase left ventricular burden, and ultimately affect diastolic function (17). In addition, although left ventricular hypertrophy (LVH) is highly correlated with LVMI, they are not completely equivalent. In this study, LVH was included as a binary variable to evaluate the independent contribution of the established pathological state of hypertrophy, whereas LVMI was included as a continuous variable to capture the continuous risk gradient associated with changes in left ventricular mass. Yu et al. (18) found that hemoglobin is a protective factor against heart failure in MHD patients, consistent with the results of this study. Hemoglobin is the vital oxygen-carrying protein in red blood cells; reduced levels indicate decreased oxygen transport capacity in the blood (19). Insufficient oxygen supply to tissues and organs like the myocardium, and myocardial hypoxia itself, can affect both systolic and diastolic function, increasing the risk of LVDD occurrence.

In the nomogram prediction model for cardiovascular events in MHD patients developed by Qin et al. (20), the highest prediction AUC reached 0.826. Wang et al. (21) constructed a nomogram prediction model for cardiac valve calcification in MHD patients based on factors such as age, sex, and systolic blood pressure, with a prediction AUC of 0.845. This study constructed a nomogram prediction model based on the aforementioned influencing factors. Validation revealed that the model's predictive performance was acceptable, with the highest prediction AUC reaching 0.922. The nomograms constructed in the aforementioned studies had AUCs lower than or close to the predictive performance of this study's model. This model, based on robust statistical analysis, provides clinical healthcare professionals with a practical predictive tool that can effectively identify high-risk LVDD patients among those undergoing MHD, offering reference information for treatment decisions and potentially improving the prognosis of MHD patients. This nomogram prediction model can also aid in allocating medical resources and planning healthcare services, particularly considering the increasing prevalence of chronic kidney disease complications and the burden they place on global health systems. However, in this study, 61 outcome events were included with 6 predictor variables, yielding an events-per-variable (EPV) ratio of approximately 10, which is at the lower acceptable limit and carries a potential risk of overfitting. The high AUC may reflect overfitting and spectrum bias, rather than truly superior predictive performance.

This study has several limitations. First, despite using robust statistical methods, the retrospective nature of this study might introduce potential case selection bias. Second, data were collected from a single center, which may limit the generalizability and applicability of the findings. Before this model can be generalized to clinical practice in other centers, external validation in multicenter prospective cohorts from different regions and under different dialysis management patterns is required. Future validation studies should pay particular attention to differences in population structure (such as age distribution, comorbidity spectrum, and dialysis vintage) as well as variations in healthcare conditions (including dialysis adequacy standards, blood pressure control targets, ultrasound equipment, and interpretation criteria), in order to assess the model's generalizability. If necessary, threshold adjustment or coefficient recalibration should be performed to facilitate eventual clinical translation. Third, this study did not consider the impact of other potential confounding factors such as medication use and lifestyle, which could also increase the risk of LVDD development. Fourth, the nomogram model was validated using data from the same center, lacking external validation. Therefore, collaboration with other centers is needed to validate the applicability of the nomogram model in a broader population. Fifth, the criteria used in this study to assess LVDD may differ to some extent from those adopted in other studies, which may contribute to variations in the reported prevalence of LVDD. Comparative analyses will be conducted in future research. Sixth, the prediction model developed in this study may be subject to overfitting and therefore requires further validation. Seventh, hematological laboratory parameters, such as hemoglobin, were measured only once in this study and may not reflect long-term status, potentially affecting the stability of the model. Future studies should consider assessing their dynamic changes to better evaluate their relationship with LVDD.

In summary, a nomogram prediction model was developed based on the influencing factors of age, left ventricular hypertrophy, hypertension, diabetes, LVMI, and hemoglobin. The development of this nomogram prediction model can be used to predict high-risk LVDD patients among those undergoing MHD, providing a reference for managing the occurrence of cardiovascular complications associated with chronic kidney disease. This nomogram tool can aid clinical decision-making and has the potential to improve patient care, especially in resource-limited settings.

## 5 Research involving human participants

This study involving human participants was in accordance with the ethical standards of the Medical Ethics Committee of Wenzhou Central Hospital and with the 1964 Helsinki Declaration. And obtain the informed consent form of the patient, and sign on the informed consent form.

## Data Availability

The original contributions presented in the study are included in the article/Supplementary Material, further inquiries can be directed to the corresponding author.
